# Testing a self-determination theory model of children’s physical activity motivation: a cross-sectional study

**DOI:** 10.1186/1479-5868-10-111

**Published:** 2013-09-26

**Authors:** Simon J Sebire, Russell Jago, Kenneth R Fox, Mark J Edwards, Janice L Thompson

**Affiliations:** 1 Centre for Exercise Nutrition and Health Sciences, School for Policy Studies, University of Bristol, BS8 1TZ Bristol, United Kingdom; 2 School of Sport and Exercise Sciences, University of Birmingham, B15 2TT, Edgbaston, Birmingham, England

**Keywords:** Motivation, Physical activity, Children, Self-determination theory, Physical activity

## Abstract

**Background:**

Understanding children’s physical activity motivation, its antecedents and associations with behavior is important and can be advanced by using self-determination theory. However, research among youth is largely restricted to adolescents and studies of motivation within certain contexts (e.g., physical education). There are no measures of self-determination theory constructs (physical activity motivation or psychological need satisfaction) for use among children and no previous studies have tested a self-determination theory-based model of children’s physical activity motivation. The purpose of this study was to test the reliability and validity of scores derived from scales adapted to measure self-determination theory constructs among children and test a motivational model predicting accelerometer-derived physical activity.

**Methods:**

Cross-sectional data from 462 children aged 7 to 11 years from 20 primary schools in Bristol, UK were analysed. Confirmatory factor analysis was used to examine the construct validity of adapted behavioral regulation and psychological need satisfaction scales. Structural equation modelling was used to test cross-sectional associations between psychological need satisfaction, motivation types and physical activity assessed by accelerometer.

**Results:**

The construct validity and reliability of the motivation and psychological need satisfaction measures were supported. Structural equation modelling provided evidence for a motivational model in which psychological need satisfaction was positively associated with intrinsic and identified motivation types and intrinsic motivation was positively associated with children’s minutes in moderate-to-vigorous physical activity.

**Conclusions:**

The study provides evidence for the psychometric properties of measures of motivation aligned with self-determination theory among children. Children’s motivation that is based on enjoyment and inherent satisfaction of physical activity is associated with their objectively-assessed physical activity and such motivation is positively associated with perceptions of psychological need satisfaction. These psychological factors represent potential malleable targets for interventions to increase children’s physical activity.

## Background

Many children are insufficiently physically active [1]. To increase children’s physical activity (PA), it is necessary to understand its social, environmental and psychological underpinnings [2]. Understanding the social cognitive factors that can be influenced by children’s social milieu is important because they could be targeted through theory-based interventions to increase PA [3]. For example, giving advice to influential figures in children’s lives such as teachers and parents on how to support PA may provide a mechanism for increasing children’s motivation.

Motivation is an individual’s drive to act, and self-determination theory (SDT) [4] is being widely applied to study PA motivation throughout the lifespan [3,5-7]. SDT contains many elements that have established relevance to PA in a single theoretical framework including personal motivation, psychological and social-environmental antecedents to motivation [3]. A multi-dimensional view of motivation is taken in SDT. Specifically, different types of motivation are arranged on a continuum based on their degree of self-determination [4]. Such a perspective focuses upon the *quality* in addition to the quantity of motivation, with self-determined (or autonomous) motivation types considered to be higher quality than less self-determined (or controlling) types of motivation. Six motivation types are proposed in SDT; intrinsic motivation, integrated regulation, identified regulation, introjected regulation, external regulation and amotivation. Intrinsic motivation is based on the inherent interest and satisfaction derived from being active rather than engaging for a separable outcome and is considered the most autonomous form of motivation. Integrated, identified, introjected and external regulations are extrinsic forms of motivation because of their instrumental focus on consequences not inherent in the activity. Integrated (i.e., where PA reflects an individual’s values and broader goals) and identified (i.e., personally valuing the benefits of being active) regulation are considered autonomous forms of extrinsic motivation. On the other hand, introjected regulation (i.e., PA participation is driven by internal pressures to avoid guilt or shame and to enhance or protect one’s ego) and external regulation (i.e., being active to obtain performance-based rewards, comply with demands/expectations or avoid punishment) are considered controlling forms of extrinsic motivation [4,6]. In contrast to these different types of motivation, amotivation is defined as an absence of motivation or intention to act [4]. Fundamental to SDT is the hypothesis that autonomous motivation is associated with positive cognitive, affective and behavioral outcomes whereas controlled forms of motivation will undermine these outcomes [4,6].

Applying SDT to investigate PA motivation is advantageous because the psychological conditions which underpin the quality of motivation are specified. Such conditions, which can be influenced by individuals’ social environments (e.g., by a child’s teacher, coach or parent), provide targets for behavioral interventions [7]. Specifically, three psychological needs are hypothesised which are considered to be psychological nutriments required for autonomous motivation and psychological well-being. The needs are, autonomy (i.e., to be choiceful and the origin of one’s action), competence (i.e., to feel effective and confident in one’s abilities and actions) and relatedness (i.e., to feel a sense of meaningful and mutual connectedness with others) [4,7].

### Physical activity motivation and behavior

Research comprising samples from a range of countries has explored the association between PA-based behavioral regulations and leisure-time PA among youth [8-10]. Similar to research exploring motivation for school physical education (PE) and PA levels within PE classes [11-13], the collective evidence from this literature suggests that more autonomous forms of PA motivation are positively associated with PA whereas controlled forms of motivation are largely unrelated to PA [8-10].

The majority of this research has relied on self-report measures of PA [8-10]. However, studies of social cognitive theories of PA need to include objective measures to address the limitations of self-report techniques such as inaccurate recall and common-method artefacts [3]. Accordingly, studies of adolescents’ motivation, have adopted more objective measures; primarily pedometry [14,15] and accelerometry [16-18]. These studies have identified small positive associations between adolescents’ composite autonomous motivation scores and pedometer step counts [14,15], intrinsic, identified and introjected PA motivation [16], a composite self-determined motivation score [18] and accelerometer-assessed PA.

Given the importance of establishing PA from an early age, it is imperative to extend this line of research to study the PA motivation of children. Within SDT, the cognitive, affective and behavioral effects of both autonomous motivation and psychological need satisfaction are hypothesised to be important throughout the lifespan [19]. However it is possible that certain forms of motivation may be more salient than others at different life stages. For example, PA during early childhood may be underpinned by intrinsically motivated play [20] or externally driven compliance with a parent’s wishes, whereas motivation for PA during adolescence may be driven more by self-identified benefits or introjected societal expectations related to body image [19].

A recent study among children examined associations between different PA motives and accelerometer-derived PA scores [21]. Competence and enjoyment motives, which are likely associated with autonomous behavioral regulation [22], showed small positive associations with PA. Although this study benefitted from measuring PA with accelerometry, the measure of motivation reflected a combination of goal content, which refers to the “what” of motivation or the specific type of goal (e.g., to develop skills) [22] and behavioral regulation (i.e., the underlying “reason why” of motivation), which are related yet theoretically distinct components of motivation [22,23]. This prevents conclusions from being drawn regarding the associations between the behavioral regulations proposed in SDT and children’s PA, which is an important research gap.

### The role of psychological need satisfaction

Previous research has also neglected to study the role of psychological need satisfaction in underpinning children’s PA motivation. The positive motivational and psychological consequences of psychological need satisfaction are supported in studies of adults’ PA [22] and adolescents’ need satisfaction and motivation within PE [24,25]. In a sample of Greek adolescents [26], small positive associations were found between competence and relatedness (not autonomy) need satisfaction in PA and autonomous PA motivation. Despite the interest in understanding children’s PA motivation from the SDT perspective, no research has considered the role of psychological need satisfaction in the motivational sequence of children’s PA. A potential reason for this lack of research may be that there are no validated questionnaires with which to measure children’s PA-based behavioral regulation or psychological need satisfaction.

In summary, there is little research investigating the associations between children’s psychological need satisfaction in PA, their motivational regulations as defined in SDT and their PA. Previous research has largely been conducted among adolescents, has adopted subjective measures of PA and there are no validated measures of motivational regulation or psychological need satisfaction for use with children. To address these limitations the present study sought to: (1) Refine and adapt existing measures of behavioral regulation and psychological need satisfaction to the context of children’s PA and examine the construct validity of scores derived from the scales, (2) examine the associations between PA behavioral regulation and objectively-assessed PA among children and (3) test a sequential theoretical model in which PA psychological need satisfaction is associated with more self-determined forms of behavioral regulation which are in turn associated with PA.

## Methods

### Sampling and participants

Data reported are from the baseline measures taken in the Action 3:30 project. Action 3:30 is a pilot randomized controlled trial of a teaching-assistant led extra-curricular PA intervention for children in years 5 and 6 of British primary schools who are not usually active through traditional or organised school sport teams [27]. Participant recruitment to the Action 3:30 feasibility trial is described elsewhere [27]. The final sample were 462 children (56.9% girls) from 20 primary schools located in the greater Bristol Area, UK. School cluster size ranged from 12 to 30 children (Mean = 23.1, SD = 5.51). Children were from Year 5 (n = 246, 53.3%) and Year 6 (n = 216, 46.7%) with a mean age of 10.03 years (SD = 0.566) and range of 7.84 to 11.09 years. The body mass index (BMI) (Mean BMI SDS = 0.529, SD = 1.145) of the participants was similar to that previously reported in UK children of this age [28]. Mean daily MVPA was 57.94 minutes (SD =20.99) and ranged from 22.67 – 149.22 minutes. Ethical approval was granted by a University of Bristol Ethics Committee, with informed parental consent obtained for all participants.

### Measures

#### Self-determined motivation for physical activity

The measurement of self-determined motivation for PA among young people has previously been limited to adolescents [11,15,16] and specific contexts such as PE [12,24]. We therefore sought to develop/adapt a set of items appropriate for the measurement of children’s intrinsic, identified, introjected and external motivational regulations for PA. The Behavioural Regulations in Exercise Questionnaire [29] served as a starting point. This scale is consistent with theoretical definitions and has shown good psychometric properties in adolescents [30]. Within SDT, integrated regulation is the most autonomous form of extrinsic motivation. In PA, integrated regulation refers to motivation derived from the alignment of PA with one’s developed sense of self and broader life goals [6]. This is an advanced form of motivation not usually displayed by children [31] and as such, we did not measure integrated regulation. Similarly as we were primarily interested in measuring the quality of children’s motivation, we did not include items assessing amotivation (which assesses the presence vs. absence of motivation rather than the level of self-determination). Items were screened individually for age appropriateness and simplifications to wording based on published measures of children’s self-determined motivation in other contexts (e.g., academic subjects) [32] and references to exercise were replaced with PA. To reduce participant burden, 12 items (3 per motivation subscale) were specified. The items were screened by three academics with expertise in children’s motivation, development and PA who provided feedback on theoretical alignment, construct coverage and item clarity.

The adapted scale consisted of the stem “*Boys and girls can be active by doing all sorts of things, for example walking, playing out, or doing sports. The following pages have some reasons why you might be active. Please indicate how true each one is for you*”. Items were preceded by “*I am active because*”. Three items each measured intrinsic (e.g., *Being active is fun*), identified (e.g., *It is important to me to do active things*) introjected (e.g., *When I’m not active I feel bad*) and external motivation (e.g., *Other people say I should be*). Items were scored using a 5-point likert-type scale: 1 (*not true for me*) to 5 (*very true for me*).

#### Physical activity psychological need satisfaction

Autonomy (e.g., *I can decide what activities and sports I want to do*) and competence (e.g., *I am happy with how good I am at doing active games*) were measured using two six-item scales from a measure used previously in a PE setting [33]. Items were adapted to fit the PA context and simplified for the age-group. Relatedness (e.g., *I am included by others in active games / sports*) was measured using an adapted version of the six-item Relatedness to Others in Physical Activity Scale [34]. Modifications to wording were made to increase item clarity for the target age group which were verified by the original scale author. Items were scored using a 5-point likert-type scale ranging from 1 (*not like me at all*) to 5 (*really like me*).

Feedback on both of the new scales was sought from two primary school teachers with regards to clarity of the items. Flesch-Kincaid Grade Level reading scores (based on average sentence length & number of syllables) indicated that the reading age was appropriate for the target age group.

#### Physical activity

Participants were asked to wear an ActiGraph (Pensacola, FL) GT3x accelerometer on an elastic waistband for five consecutive days including two weekend days. Accelerometers recorded in 10 second epochs. Data were downloaded to ActiLife software and processed using KineSoft Software (Version 3.3.62). Periods greater than an hour with zero values were considered non-wear time and were removed. Only data from children with ≥ 3 valid days (i.e., ≥480 mins) were analysed. Mean minutes of moderate-to-vigorous PA (MVPA) per valid day was derived using a cut-point appropriate for children [35]. 47.4% of participants provided five valid days of accelerometer data, 29.9% provided four valid days, and 22.7% provided three valid days. The mean length of a valid day was 733.33 minutes (SD = 68.58) (12.22 hours). This sample (N = 462) represented 86% of the total Action 3:30 baseline sample. Participants who did not provide valid data (n = 73) reported greater external PA motivation (*t* (95.50) = 2.378, *p* = .01) and comprised a greater proportion of boys (57% vs. 43%, χ^2^ = 5.287, *p* = .021) compared with the children who provided valid accelerometer data.

### Data analysis

Two confirmatory factor analyses (CFA) were performed in Stata 12 to assess the construct validity of the factor structure of the four motivation and three psychological need satisfaction scales. For both scales, maximum-likelihood estimation was used and the chi-square statistic, confirmatory fit index (CFI), root mean square error of approximation (RMSEA) and standardized root mean square residual (SRMR) were analysed to assess model fit [36]. For the CFI, thresholds of > .90 and > .95 are indicative of acceptable and excellent fit respectively between the model and data and values of ≤ .06 and ≤ .08 were deemed indicative of a well-specified model for the RMSEA and SRMR respectively [36]. Modification indices were analysed in combination with theoretical postulations to identify areas for model respecification [37].

To account for clustering of participants within schools, clustered standard errors were analysed. The PA motivation CFA model was specified to consist of four co-varying latent factors (i.e., intrinsic, identified, introjected and external motivation), each defined by three observed factors (questionnaire items). The psychological need satisfaction scale CFA model consisted of three co-varying latent factors (i.e., autonomy, competence & relatedness) each defined by six observed factors. Following CFA and model re-specification, factor correlations, means, SDs and internal consistency estimates (Cronbach α) were calculated for all scales. Bivariate correlations between each of the psychological variables and MVPA were derived.

**Figure 1:**
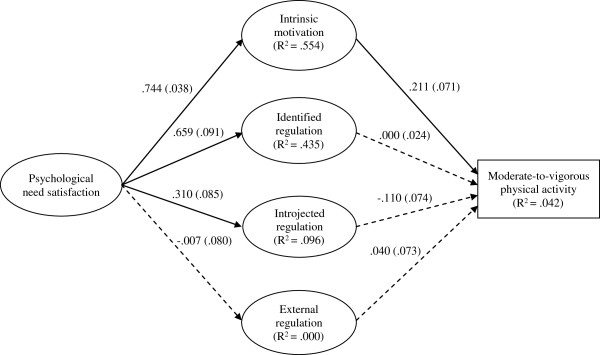
**Sequential model of motivation predicting moderate-to-vigorous physical activity of primary school children.** Note: Parameter estimates are standardized. Solid arrows represent significant estimates (all p <.003) and dashed arrows represent non-significant estimates (p>.05). Values in parentheses are robust standard errors. Co-variances between disturbance terms of theoretically-related motivation variables were: Cov_intrinsic-identified_ = .53 (.147) p <.001; Cov_introjected-external_ = .68 (.054) p <.001.

Structural equation modelling in Stata 12 was used to analyse the proposed model of motivation. To achieve an acceptable participant to estimated parameter ratio [38], a latent variable representing psychological need satisfaction was specified, defined by three need satisfaction observed variables (autonomy, competence & relatedness). Psychological need satisfaction preceded the four latent variables representing the motivation types, each defined by three observed items. Finally, paths were specified leading from each motivation type to an observed variable representing minutes of MVPA (Figure 1). Due to positive skewness, the MVPA variable was square-root transformed. With 16 observed variables, the model was over-identified and the participant-to-estimated parameter ratio was approximately 10:1 which was sufficient [38]. Maximum-likelihood estimation was used and model fit was assessed using the fit indices and criteria adopted in the CFAs. Similarly, to account for clustering of participants within schools, robust standard errors were used.

## Results

### Construct validity of SDT-based measures

#### Physical activity motivation scales

The results of the CFA are shown in Table 1. There was an excellent fit of the initial model to the data. Standardized item-factor loadings were all significant (p < .001) and ranged from .451 to .786. The bivariate correlation matrix (Table 2) supported a simplex-pattern and theoretical tenets in which motivation types more proximally located on the motivational continuum (i.e., intrinsic-identified) were more strongly associated than those more distally positioned (i.e., intrinsic-introjected). Cronbach’s alpha coefficients for the motivation subscales were: intrinsic (α = .77), identified (α = .71), introjected (α = .59) and external (α = .71). Individual standardized item loadings and factor correlations are presented in Additional file 1: Table S1.

**Table 1:** Results of confirmatory factor analysis of motivation and psychological need satisfaction scales

	**χ**^**2 **^**(df), p**	**CFI**	**SRMR**	**RMSEA (90% CI)**
Physical activity behavioral regulation scale				
Model 1	74.58 (46), p =.005	.981	.032	.037 (.020, .051)
Physical activity psychological need satisfaction scale				
Model 1	316.96 (132), p <.001	.929	.044	.055 (.047, .063)
Model 2	255.74 (101), p <.001	.940	.041	.057 (.049, .066)
Model 3	255.75 (99), p <.001	.951	.040	.052 (.040, .060)

Note. CFI = comparative fit index; SRMR = standardised root mean square residual; RMSEA = root mean square error of approximation.

**Table 2:** Bivariate correlations among children’s (N = 462) motivation, psychological need satisfaction and physical activity

		***M***	***SD***	**1**	**2**	**3**	**4**	**5**	**6**	**7**	**8**
1.	Intrinsic motivation	4.514	.648	-							
2.	Identified regulation	4.255	.742	.567	-						
				(<.001)							
3.	Introjected regulation	3.254	.929	.247	.379	-					
				(<.001)	(<.001)						
4.	External regulation	2.423	1.050	.029	.169	.494	-				
				(.539)	(<.001)	(<.001)					
5.	Autonomy need satisfaction	4.112	.602	.548	.472	.194	-.061	-			
				(<.001)	(<.001)	(<.001)	(.191)				
6.	Competence need satisfaction	3.868	.669	.448	.363	.243	-.011	.585	-		
				(<.001)	(<.001)	(<.001)	(.818)	(<.001)			
7.	Relatedness need satisfaction	4.029	.726	.419	.364	.246	.077	.534	.562	-	
				(<.001)	(<.001)	(<.001)	(.099)	(<.001)	(<.001)		
8.	Moderate-to-vigorous PA (min per day)	57.937	20.993	.174	.031	.002	.016	.128	.139	.099	-
				(<.001)	(.505)	(.966)	(.726)	(.006)	(.003)	(.033)	

Note. Values in parentheses are *p*-values.

#### Psychological need satisfaction scales

The results of the CFA are shown in Table 1. There was a good fit of the model to the data however two items (one competence & one autonomy) displayed low factor loadings with weak evidence against null item-factor associations and were therefore removed. This re-specification (Table 1, model 2) improved model fit. Modification indices identified that allowing the error terms of three of the items assessing relatedness would improve model fit. As the items were indicators on the same theoretically-derived subscale, this modification was made (Table 1, model 3) and an improved fit was observed. Standardized factor loadings ranged from .586 to .748. Correlations between the subscales (Table 2) indicated that the need satisfaction variables were positively correlated as expected in SDT. Cronbach’s alpha coefficients were as follows: autonomy (5 items) α = .72; competence (5 items) α = .82 and relatedness (6 items) α = .81. Individual standardized item loadings are presented in Additional file 1: Table S1.

### Motivation model

The results of the initial SEM indicated a poor fit of the model and the data: χ^2^ (99) = 461.428, p < .001; CFI = .839; SRMR = .093; RMSEA = .089 (90% CI = .081 to .097). Examination of modification indices suggested that permitting two error co-variances (i.e., between intrinsic motivation and identified regulation and between introjected and external regulation) would improve model fit. As these co-variances align with the theoretical distinction between autonomous (intrinsic and identified) and controlled (introjected and external) types of motivation these modifications were made. The re-specified model resulted in an improved fit: χ^2^ (98) = 314.05, p < .001; CFI = .904; SRMR = .068; RMSEA = .069 (90% CI = .061 to .078) (Figure 1). Examination of the standardised paths showed that need satisfaction was positively associated with intrinsic identified and introjected motivation and un-related to external motivation. Intrinsic motivation displayed a positive association with MVPA and shared approximately 4% of the variance with MVPA scores. To investigate the associations between individual autonomy, competence and relatedness need satisfaction factors and intrinsic motivation, a further structural equation model was analysed in which paths were specified between the three need satisfaction constructs and intrinsic motivation which in turn was associated with MVPA. The model fit was as follows [χ^2^ (df) = 373.77 (164), p < .001; CFI = .935; SRMR = .044; RMSEA = .053 (90% CI = .046 to .060)]. The parameter estimates indicated that autonomy need satisfaction was strongly positively associated with intrinsic motivation (p < .000) (Additional file 2: Figure S1). Competence and relatedness need satisfaction were not associated with intrinsic motivation.

## Discussion

The data presented here provide preliminary evidence for the construct validity, internal consistency and theoretical alignment of the revised behavioral regulation and need satisfaction scales among primary school-aged children. Bivariate correlations among the motivation subscales supported theoretical tenets of a simplex-like ordering of motivation types on a self-determination continuum [4]. The internal consistency of the three need satisfaction scales and three of the four motivation subscales was acceptable; however the internal consistency of the introjected motivation items was below accepted thresholds [39]. Previous research has reported low internal consistency of scales measuring introjected PA motivation among children [14] and adolescents [16]. However, other research has found measures of introjected regulation within PE and exercise to be internally consistent [15].

The measurement of introjected PA regulation among children is challenging as it requires participants to understand and recognise feelings of guilt and shame as a source of motivation. Additionally, participants are required to differentiate introjected motivation from external motivation, the latter of which may be clearer. Introjection may be too abstract for young children, relying on more advanced cognitive / self-perception development. In line with the perspective that children develop more differentiated self-perceptions at approximately 8 years of age [40] previous studies measuring motivation of younger children (i.e., grades 1–3) have combined introjected and external types into a single controlled motivation indicator [32]. However, in the present study, we rephrased PA introjection items to reflect *feeling bad* about oneself if not active and *showing other people how good I am* which are likely understood by 9–11 year olds [40]. Further, introjected and external regulation were only moderately correlated suggesting children could differentiate between these scales. Future work could examine the utility of measuring introjected and external regulation using separate and combined scales among children.

The central aim of the present work was to explore the associations between the motivation types forwarded in SDT and objectively-assessed PA among children. Intrinsic motivation was positively associated with MVPA and shared approximately 4% of its variance with the variance of accelerometer scores. This association is consistent with previous work among adolescents where PA was measured objectively [16] and by self-report [9]. The magnitude of shared variance between intrinsic motivation and PA is greater than that previously reported between adolescent’s autonomous motivation for exercise and their pedometer scores (1%) [15] although it still remains small.

Intrinsic motivation was the only type of motivation associated with children’s PA behavior. This suggests that interventions to increase children’s PA should be designed to optimise children’s enjoyment of PA and ensure that they can find inherent satisfaction in being active rather than relying on forms of extrinsic motivation. Designing interventions that achieve this will require substantial formative research and participant engagement to ensure enjoyment is achieved and maintained [41]. Previous research among adolescents has also reported positive associations between identified regulation and PA [9,16]. The comparative lack of an association in our findings may indicate that internalised extrinsic forms of motivation for PA (e.g., improvements in health) are more central to adolescent’s PA engagement than they are for children.

Lending support to the tenets of SDT that controlling forms of motivation do not underpin meaningful behavioral engagement [4], both introjected and external regulation were unrelated to children’s PA in the present study. Previous research among adolescents has identified inconsistent cross-sectional associations between introjected regulation and PA [9,16]. These findings again point to potential developmental differences in PA motivation and highlight the importance of considering children’s and adolescent’s motivation separately. It is important to reiterate that the introjected motivation subscale had low internal consistency and as such, results need to be interpreted with caution.

Within SDT, more autonomous forms of motivation are hypothesised to be underpinned by the satisfaction of needs for autonomy, competence and relatedness [4]. In the present work, a need satisfaction latent factor explained substantial proportions of variance in intrinsic motivation (55%) and identified regulation (44%). The magnitude of these associations is similar to those found between need satisfaction and motivation in PE lessons [25] and the findings extend previous work in youth PA settings which found that only relatedness and competence needs were weakly associated with an autonomous motivation composite score [26]. Supplementary analysis showed that the association between need satisfaction and intrinsic motivation was primarily carried by perceptions of autonomy need satisfaction. This underpins the importance that children’s social environments (e.g., teachers, parents, coaches) foster perceptions of choice, freewill and volition in order to develop their intrinsic motivation towards PA. While in the bivariate analysis both competence and relatedness were positively and moderately associated with intrinsic motivation, the lack of association between competence and relatedness and intrinsic motivation in the multivariable structural model is not consistent with SDT. A potential explanation is the relatively large factor correlations observed among the need satisfaction subscales. Future work is needed to tease out in more detail how children distinguish between and verbalise the three need satisfaction constructs and to optimise measures to reflect this.

Due to sample size restrictions and our primary focus on exploring the associations between individual motivation types and MVPA, we did not explore associations between individual needs and all behavioral regulations. However, bivariate correlations largely supported SDT, provided further evidence for the validity of the adapted scales and illuminated PA motivational processes among children. Specifically, all three needs displayed moderate-to-strong correlations with identified regulation. Need satisfaction was not correlated with external regulation in either bivariate or SEM analyses. Small-to-moderate correlations were found between all needs and introjected regulation. Such findings are contrary to SDT, but similar associations have been found in the PE motivation literature [25,33]. In the present study, the need satisfaction-introjected regulation association was much weaker than the association between need satisfaction and both types of autonomous motivation. Further, the shared variance was approximately 10%, lower than for intrinsic motivation and identified regulation.

This unexpected association could reflect the unique nature of need satisfaction and introjection in PA [33]. Specifically, because much of children’s PA is likely to be enacted in social contexts with friends or siblings, children may have interpreted items about *feeling bad when not being active* as feelings related to disappointing their friends / siblings (e.g., if not able to play out), thereby reflecting a strong connection with active others which was assessed by the relatedness items (i.e., *others want me to be active with them*). The discordance between theory and empirical findings with regards to need satisfaction and introjected motivation among children and adolescents warrants further investigation.

The findings presented here suggest that psychological need satisfaction is a potential route to autonomous motivation among children. From the SDT perspective, psychological need satisfaction in PA can be manipulated by social agents (teachers, parents, coaches) either adopting autonomy-supportive (need satisfying) or controlling (need thwarting) interpersonal communication strategies [6]. As children’s PA is often facilitated by an adult and such social environmental factors are particularly salient, the psychological needs represent clear targets for interventions seeking to increase children’s autonomous motivation and develop their long-term competence, active friendship groups and enjoyable PA experiences.

### Limitations and future directions

While the scales adapted and tested provide theoretically coherent tools with good preliminary evidence of construct validity, scale development is an ongoing process [42], and there is scope to further test and validate scores derived from the motivation and need satisfaction measures. Particular attention could be given to further test and improve the internal consistency of the introjection items. In addition, in order to reduce participant burden we did not include a measure of amotivation. As such, the motivation measure assumes a presence of motivation, albeit of varying *quality* (i.e., self-determination) and does not represent children with low *quantity* motivation (i.e., amotivated). Future work could develop / adapt amotivation items and test a more complete motivational continuum. Central to scale validation is testing the invariance of scale structures between different populations (e.g., children of different ages, genders, ethnicities). We were prevented from doing so due to the sample size of subgroups and future research should seek to do this.

A limitation of the motivation model tested is that it did not include indicators of environmental support for the children’s PA. Previous research suggests that PE teacher autonomy support is associated with need satisfaction [33] and measures of parent, teacher and friend autonomy support [8] could facilitate the testing of these associations in the PA context. A further limitation of the tested model was the specification of a composite need satisfaction variable which prevented the examination of associations between the individual need satisfaction variables and each behavioral regulation. Future studies with larger samples are needed to examine this.

Although the Action 3:30 project aimed to recruit children who were not physically active through traditional or after-school sports, the participants were relatively physically active, on average achieving slightly less than 60 minutes of MVPA per day. In addition, participants who complied with the accelerometer data collection protocol reported lower external motivation than children who did not provide valid accelerometer data. It is important to examine motivational models among less active and poorly motivated children as these are arguably the most important to target in interventions. Finally, the data examined were cross-sectional and although causality is inferred by theoretical propositions, the data do not provide evidence for the direction of associations within the motivational model. Previous research supports prospective associations within similar models [15] and future research is required to examine such relationships within children’s PA motivation.

## Conclusions

Children’s intrinsic motivation was associated with their objectively-assessed PA. Identified, introjected and external forms of motivation were not associated with PA. The findings suggest that interventions for children which focus on having fun while being active, and creating inherently satisfying and enjoyable PA opportunities are more likely to lead to PA behavior than interventions based on educating children about the benefits of being active. Psychological need satisfaction (in particular autonomy) in PA was strongly associated with intrinsic motivation and identified regulation, suggesting possible malleable factors that could be targeted in interventions aimed at increasing children’s PA. The study provides preliminary evidence for the validity of scores derived from theoretically coherent scales adapted to measure the children’s motivation and psychological need satisfaction towards PA.

## Abbreviations

BMI SDS: Body mass index standard deviation score; CFA: Confirmatory factor analysis; CFI: Comparative fit index; MVPA: Moderate-to-vigorous physical activity; PA: Physical activity; PE: Physical education; RMSEA: Root mean square error of approximation; SDT: Self-determination theory; SEM: Structural equation modelling; SRMR: Standard root mean residual

## Competing interests

The authors declare that they have no competing interests.

## Authors' contributions

RJ & SJS conceived of the Action 3:30 project. SJS conceived of the paper, refined/amended the psychological measures, performed the analysis, drafted the manuscript and led the revisions. KRF assisted with refinement of the psychological measures. MJE coordinated all data collection. JLT assisted with interpretation. All authors made critical academic contributions to the manuscript and read and approved the final version.

## Supplementary Material

Additional file 1: Table S1 Confirmatory factor analysis results for measures of self-determined motivation and psychological need satisfaction among children (n=462). Click here for file

Additional file 2: Figure S1 Structural equation model of associations between psychological need satisfaction, intrinsic motivation and physical activity of primary school children. Click here for file
