# Supplementary material for: Testing a self-determination theory model of children’s physical activity motivation: a cross-sectional study
Source: Int J Behav Nutr Phys Act. 2013 Sep 26;10:111. doi: 10.1186/1479-5868-10-111 (PMC3852537; doi:10.1186/1479-5868-10-111)
Supplement: Additional file 1: Table S1 — Confirmatory factor analysis results for measures of self-determined motivation and psychological need satisfaction among children (n=462). [file 1479-5868-10-111-S1.pdf]

Additional file 1: Table S1: Confirmatory factor analysis results for measures of self-determined motivation and psychological need satisfaction among children (n=462).

	Standardized loading	95% CI	Standard error*	P
**Physical Activity Motivation**				
**Intrinsic**				
Being active is fun	**.673**	**.596, .751**	**.039**	**.000**
I enjoy being active	**.786**	**.726, .847**	**.031**	**.000**
I like being active	**.751**	**.646, .857**	**.054**	**.000**
**Identified**				
It is important to me to be active	**.710**	**.651, .769**	**.030**	**.000**
I value the benefits of being active	**.622**	**.542, .702**	**.041**	**.000**
In life it is important to be active	**.669**	**.587, .751**	**.042**	**.000**
**Introjected**				
When I'm not active I feel bad	**.451**	**.342, .560**	**.056**	**.000**
When I don't do activity I feel bad about myself	**.515**	**.389, .641**	**.064**	**.000**
I want to show other people how good I am	**.451**	**.331, .570**	**.061**	**.000**
**External**				
Other people say I should be	**.658**	**.587, .729**	**.036**	**.000**
**If** I'm not, other people will not be pleased with me	**.685**	**.600, .769**	**.043**	**.000**
Other people pressure me to be active	**.656**	**.549, .762**	**.054**	**.000**
**Factor Correlations (p-value)**	**Intrinsic**	**Identified**	**Introjected**	**External**
**Intrinsic**	-			
**Identified**	**.755 (.000)**	-		
**Introjected**	**.402 (.000)**	**.701 (.000)**	-	
**External**	**.031 (.642)**	**.233 (.000)**	**.880 (.000)**	-
**Psychological Need Satisfaction**				
**Autonomy**				
I can decide which activities / sports I want to do	.606	.499, .714	.055	.000
I have a say in what activities / sports that I want to do	.602	.505, .698	.049	.000
I feel I am active because I want to be	.606	.522, .689	.043	.000
I feel free when I'm active	.544	.432, .655	.057	.000
I have some choice in what activity / sport I want to do	.603	.511, .694	.047	.000
**Competence**				
When it comes to playing active games, I think I am pretty good	.740	.681, .799	.030	.000
I think I do well, compared to other children my age	.682	.601, .764	.042	.000
After working at a new activity for a while, I feel that I can do it pretty well	.619	.531, .707	.045	.000
I am happy with how good I am at doing active games	.682	.627, .737	.028	.000
When it comes to being active, I think I have good physical skills.	.748	.682, .813	.033	.000
**Relatedness**				
I am included by others	.627	.525, .729	.052	.000
I feel like I'm part of a team	.678	.601, .755	.040	.000
I am supported by others	.711	.676, .745	.018	.000
Others want me to be active with them	.632	.559, .704	.037	.000
I have close bonds with others	.586	.493, .679	.048	.000
I fit in well with others	.672	.589, .756	.043	.000
**Factor Correlations (p-value)**	**Autonomy**	**Competence**	**Relatedness**	
**Autonomy**	-			
**Competence**	**.839 (.000)**	-		
**Relatedness**	**.756 (.000)**	**.749 (.000)**	-	

*Robust standard errors are reported as participants were clustered within schools.
