# Supplementary material for: Testing a self-determination theory model of children’s physical activity motivation: a cross-sectional study
Source: Int J Behav Nutr Phys Act. 2013 Sep 26;10:111. doi: 10.1186/1479-5868-10-111 (PMC3852537; doi:10.1186/1479-5868-10-111)
Supplement: Additional file 2: Figure S1 — Structural equation model of associations between psychological need satisfaction, intrinsic motivation and physical activity of primary school children. [file 1479-5868-10-111-S2.pdf]

Additional file 2: Figure S1.

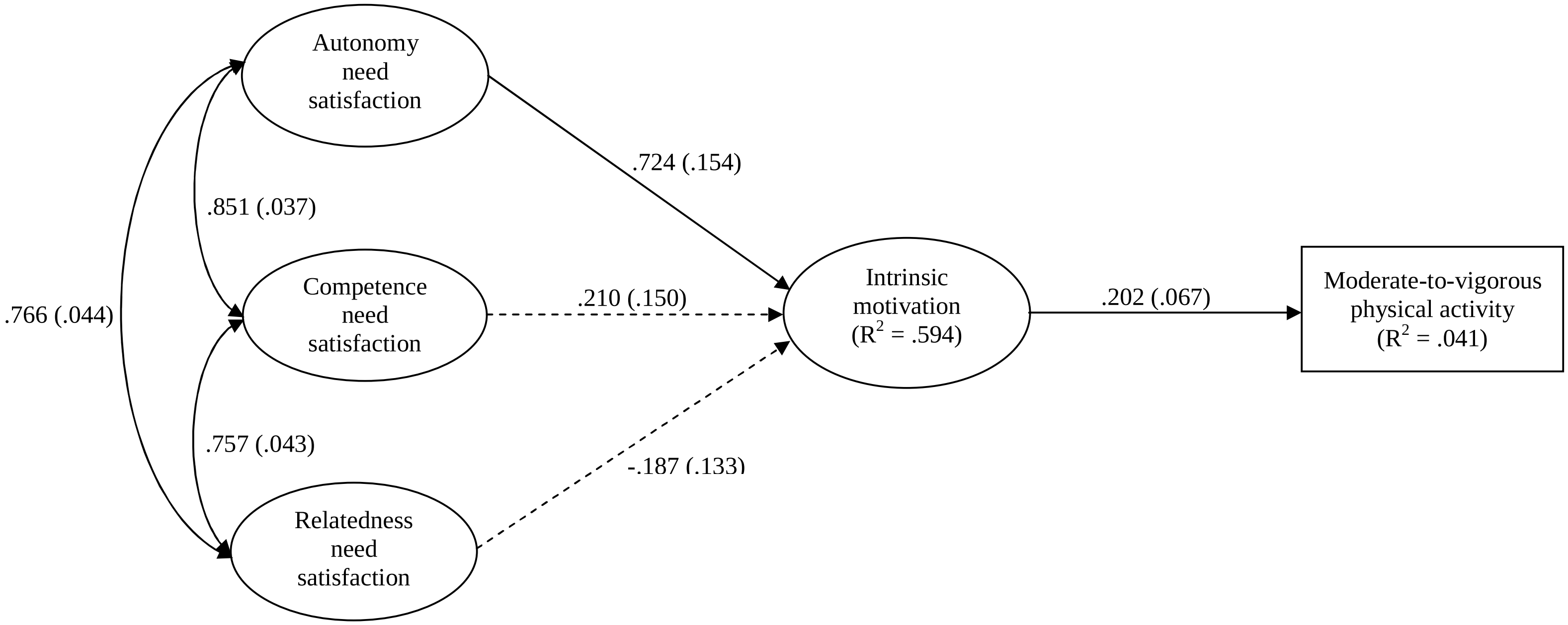

Note: Parameter estimates are standardized. Solid arrows represent significant estimates (all p <.003) and dashed arrows represent non-significant estimates (p >.05). Values in parentheses are robust standard errors.
